# EXITitis in the UK: Gravity Estimates in the Aftermath of Brexit

**DOI:** 10.1007/s10645-023-09421-3

**Published:** 2023-04-29

**Authors:** Steven Brakman, Harry Garretsen, Tristan Kohl

**Affiliations:** Faculty of Economics and Business, University of Groningen, Nettelbosje 2, 9747 AE Groningen, The Netherlands

**Keywords:** Brexit, Economic integration, Regions, Gravity model, Trade policy, F13, F14

## Abstract

The withdrawal of the United Kingdom from the European Union has had disruptive effects on international trade. As part of its ‘Global Britain’ strategy in the wake of Brexit, the UK is pursuing a series of Free Trade Agreements with countries around the world, including Canada, Japan, Korea, Mexico, Norway, Switzerland, Turkey and possibly the United States. Closer to home, the UK is under mounting pressure to dissuade Scotland, Northern Ireland and Wales from seeking independence to regain the severed ties with the EU. We analyze the economic consequences of these scenarios with a state-of-the-art structural gravity model for major economies around the world. We find that ‘Global Britain’ yields insufficient trade creation to compensate for Brexit-induced trade losses. Our results also reveal that secession from the UK in itself would inflict greater post-Brexit economic harm on the devolved nations of Great Britain. Nevertheless, these effects could be offset when secession from the UK is combined with regained EU membership.

## Introduction

As predicted by many trade studies that were published between the Brexit referendum in June 2016 and the departure by UK from the EU in January 2020 (for an overview, see Brakman et al., 2018), the actual withdrawal of the United Kingdom from the European Union has indeed had disruptive effects on international trade. Leaving the impact of the COVID19-crisis on international trade aside, Brexit has in particular harmed international trade for the UK itself; the UK government notes that trade with the EU fell by 21% and with the rest of the world 0.8% (see Sect. 2 below).^1^ As part of its ‘Global Britain’ strategy and as a reaction to its Brexit decision, the UK government is pursuing a series of Free Trade Agreements with countries around the world, including Canada, Japan, Korea, Mexico, Norway, Switzerland, Turkey and possibly the United States. Yet, closer to home, the UK is under mounting pressure to dissuade Scotland, Northern Ireland and Wales from seeking independence to regain their severed ties with the EU.

We analyze the economic consequences of these scenarios with a state-of-the-art structural gravity model for major economies around the world. We find, consistent with earlier findings, that ‘Global Britain’ yields insufficient trade creation to compensate for Brexit-induced trade losses. Our results also reveal that independence from the UK in itself would inflict greater post-Brexit economic harm on the devolved nations that are now part of Great Britain. However, these effects would be more than compensated, conditional on a renewed trade deal with the EU. In a similar vein, Huang et al. (2021) find that the costs of Scottish secession would be negative and, different from our estimates, that these cannot be compensated by rejoining the EU. It is often said that economists hardly agree on anything, but when it comes to predictions on the impact of Brexit on international trade and in particular on UK-EU trade, there was and continues to be an overwhelming consensus that Brexit has detrimental trade effects.

To date the most comprehensive, general equilibrium, study of the possible (trade) impact of Brexit on the UK economy, covering 31 sectors and aggregates and 35 regions, is Dhingra et al. (2017)^(2)^. Brakman et al. (2018) provide gravity-based estimations of the impact of Brexit on international trade, and also discuss alternative trade agreements that may arise in the wake of Brexit. Estimates on the effects of Brexit differ also because of different methodologies; some used general equilibrium approaches (Dhingra et al., 2017), others gravity models (HM treasury, 2016, or Brakman et al., 2018) and recently synthetic control techniques to reveal GDP gaps following the Brexit (Born et al., 2019). The actual numbers differ, but the vast majority of studies conclude that the impact of Brexit on the UK economy, both in terms of trade and GDP, will be a very negative one. Estimates regarding GDP range from − 2% to close to − 10%. For trade the range is a factor 2 to 3 higher, that is, trade is reduced by 4% or even more than 20%. A key outcome of the scenario studies is that alternative future trade agreements cannot make up for this impressive negative effect of Brexit. Trade agreements can soften the Brexit blow, but not make it undone. This has, for example, been found by Dhingra et al. (2017) and Brakman et al. (2018), and has recently been confirmed by Huang et al. (2021).^2^

In the present paper we will not extensively survey this literature (again), but focus instead on new estimations by employing a state-of-the-art gravity model and by using the most recent data to investigate in more detail the trade impact for alternative trade arrangements and focus on the trade consequences for *intra*-national trade for the UK economy.^3^ The latter is novel in the Brexit literature and has been under-researched until now. Partly inspired by Brexit itself, the UK faces the prospect of a break-up given the discussion of devolution or even outright independence in notably Scotland but also Wales and (Northern) Ireland. The discussion about devolution in the UK does not only relate to the economics literature because of Brexit, but it also speaks to the ‘optimum size of nations’ literature following the seminal study by Alesina and Spolaore (2003). In addition, studying the intra-UK consequences of Brexit and of alternative internal UK ‘break up’ scenarios is inspired by a growing literature linking the (the regional dispersion in the) Brexit vote or preferences to regional differences in demographic, economic, political or even psychological make-up of UK regions [see for instance Gutiérrez-Posada et al. (2021), Los et al. (2017), De Ruyter et al. (2021), Garretsen et al. (2018), respectively].

The remainder of this paper is structured as follows. Section 2 first illustrates what happened to actual UK trade now that Brexit has come into effect. A key difference with previous Brexit studies is that Brexit has materialized and we can do a first check what happened to UK trade from 2020 onwards, allowing for the fact that to date the COVID-19 crisis complicates a pre- and post-Brexit comparison. Section 3 subsequently discusses the gravity model methodology employed as well as the five trade scenarios that we will consider. In Sect. 4 we introduce and review the dataset. Section 5 presents our main estimation results, followed by an overall summary and conclusion in Sect. 6.

## Actual UK Trade After Brexit in Times of COVID-19

The UK’s EU membership formally expired on February 1, 2020—just weeks before the COVID-19 pandemic induced an unprecedented decline in world trade as of March 2020. Unfortunately, these two simultaneous trade shocks complicate the matter of empirically estimating the ex-post effects of Brexit on international trade. Yet, a comparison between the UK’s trade developments and those of similar (advanced, industrialized) nations may be insightful to obtain a *ballpark* estimate how Brexit, as a country-specific trade shock, has affected UK trade above and beyond the *global* trade shock induced by the COVID-19 pandemic. Assuming that the 2020 decline in observed world trade for various advanced, industrialized nations is largely related to the pandemic, we simply assume that any difference in the UK trends with those of other countries is an approximate measure of Brexit-induced trade effects. This calculation only serves to get a rough impression of the relative sizes of the two events.

**Fig. 1:**
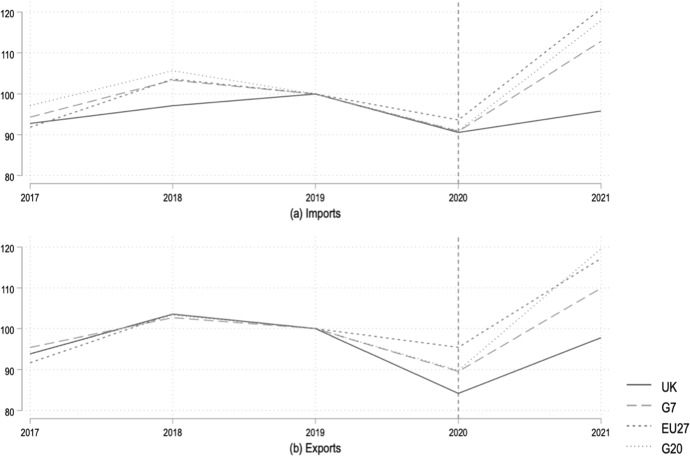
Annual aggregate trade in goods (2019 = 100). *Source*: Authors’ calculations based on IMF (2022). EU27, G7 and G20 exclude trade with the UK. G7 and G20 include France, Germany and Italy but exclude other EU27 members

**Fig. 2:**
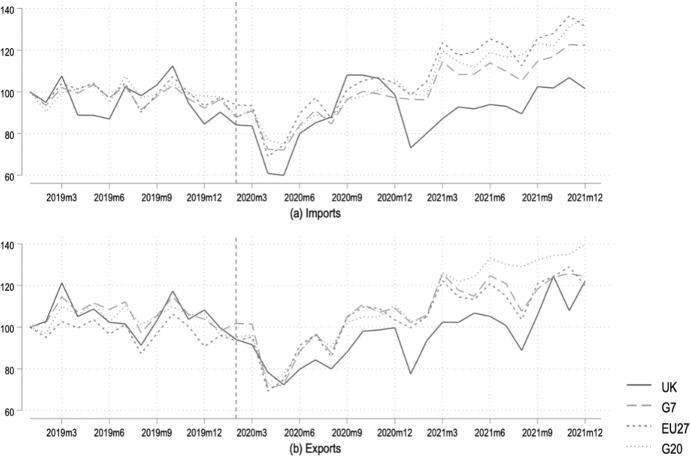
Monthly aggregate trade in goods (2019m1 = 100). Dashed vertical line reflects the beginning of the Withdrawal Agreement. *Source*: Authors’ calculations based on IMF (2022). EU27, G7 and G20 exclude trade with the UK. G7 and G20 include France, Germany and Italy but exclude other EU27 members

Figure 1 plots the development of aggregate monthly imports (top panel) and exports (lower panel) of goods for the United Kingdom, the G7 (i.e., Canada, Germany, France, Italy, Japan, United States, excluding the UK), the G20 (excluding the UK) and the EU27.^4^ Given that the pandemic was already underway during late 2019 and early 2020, we set the base month in January 2019 and observe fairly consistent trends for the UK and the peer groups under consideration. We see imports and export gradually declining for all selected units of observation, with a sudden, significant decline in trade around March–April 2020 following several lockdown policies enforced in many countries around the world. Figure 2 shows the EU27s imports dropping to a low of only 69% of the base month’s trade in April 2020; for the G7 and G20 this was around 75% in the same month. The UK’s lowest point was around the same time at just shy of 60% of its January 2019 imports, and 72% of its exports. For the EU27 and G20 exports were down, at most, to around 70% of their baseline levels.

Interestingly, aside from this example of when the pandemic seemed to have had its worst effect on international trade, the longer time horizon reflects a lower trend line for the UK in general. This observation suggests that the UK’s trade performance lags behind that of its peers—even before the official UK withdrawal from the EU—and gives us a simple proxy to distinguish pandemic-induced and Brexit-induced trade effects.

For every month after January 2019 until June 2021, we calculate the difference between the UK’s level of imports (exports) vis a vis those of the EU27, G7 and G20 (with all values to 100 in January 2019). We interpret this difference as the Brexit-induced trade effect. We then calculate the average of these monthly differences between the UK and EU27, G7 and G20 respectively, to arrive at the values presented in Table 1.

**Table 1:** Average difference between UK and selected groups of countries’ trade in goods (values in percentage points)

Frequency	EU27		G7		G20	
	Imports	Exports	Imports	Exports	Imports	Exports
Annual	− 3.1	− 11.3	− 0.3	− 5.4	− 0.4	− 5.7
Monthly	− 10.5	− 6.6	− 8.6	− 7.2	− 13.8	− 10.9

Calculations with annual data based on 2019–2021 (2019 = 100). Calculations with monthly data based on 2019m1–2021m12 (2019m1 = 100). Values are the post-2019 averages of the differences between the plotted lines for the UK and selected country groups in Figs. 1 and 2. EU27, G7 and G20 exclude trade with the UK. G7 and G20 include France, Germany and Italy but exclude other EU27 members

Table 1 shows that in terms of *annual* trade, the UK saw its imports decrease in excess of the EU27’s by 3.1 percentage points (pp) and exports by 11.3 pp. Compared to the G7, UK imports were lower by an additional 0.3 pp and exports by 5.4 pp, respectively. Compared to the G20, these differences were 0.4 and 5.7 pp, respectively. The monthly data also reflect the UK lagging behind its peer groups in terms of trade performance.

Of course, this modest attempt at disentangling the true impact of Brexit from that of the pandemic on trade is subject to several limitations. Aside from simultaneity bias, there is no theoretical guidance on whether the COVID-19 pandemic would affect the UK in either the same or in systematically different relative to the peer groups under consideration. Moreover, statistical data aggregated across several countries in the case of the EU27, G7 and G20 might mask underlying volatility in trade flows. Nevertheless, it is remarkable that the UK seems to consistently underperform relative to its peer groups. Taken together, our stylized statistics suggest that the UK experienced a sizeable negative Brexit-induced trade shock in addition to the worldwide decline in trade caused by the COVID-19 pandemic.

## Methodology: the Gravity Model Approach

We use a structural gravity trade framework to measure the effects of Brexit on international trade flows. Gravity models are the workhorse models to estimate the impact of trade policies on trade. The key idea of the model is borrowed from the Newtonian gravity model and was introduced to economics by the Dutch Economist, Jan Tinbergen (see for the history of the model van Bergeijk and Brakman, 2010). The equation states that the bigger the two countries are, the larger bilateral trade is, and that the larger the distance between two countries is, the smaller bilateral trade. These two opposing forces—country size and distance—thus determine the size of bilateral trade flows. In practice, distance is a broad concept that can describe trade barriers in general and includes variables that describe whether countries have a common border, share a similar language, had former colonial ties, are members of a Free Trade Agreement (FTA), etc.

Our calculations are based on the procedure outlined by Anderson and Yotov (2016) and Yotov et al. (2016) and follows earlier applications in Brakman et al. (2018) and Kohl (2019). The objective is to estimate the general equilibrium impact on international trade resulting from a counterfactual scenario, and compare this counterfactual outcome to the results of a baseline situation before such a counterfactual scenario is introduced. In particular, we first estimate:

1 $$\begin{aligned} X_{odt}^{BSL} & = {\mathbf{exp}}\left[ {\beta_{1} lnDST_{od} + \beta_{2} CTG_{od} + \beta_{3} INT_{od} + \beta_{4} PSA_{odt} + \beta_{5} FTA_{odt} + \beta_{6} EIA_{odt} } \right. \\ & \quad \left. { + \beta_{7} CU_{odt} + \gamma_{ot} + \delta_{dt} + \zeta_{od} } \right] \times \varepsilon_{odt} , \\ \end{aligned}$$

where *X* represents aggregate trade between origin (exporter) *o* and destination (importer) *d* in year *t*; *lnDST* is the log geographic distance in kilometers between the origin and destination, *CTG* a binary variable indicating if the country-pair shares a common border, and *INT* is a binary variable accounting for intra-national vs. international trade. We account for the heterogeneous design and impact of trade agreements by distinguishing between Partial Scope Agreements (PSA), Free Trade Agreements (FTAs), Economic Integration Agreements (EIAs), and Customs Unions (CUs); all of these binary variables are unity for country-pairs with the relevant trade agreement in a given year and 0 otherwise.^5^ Origin-year (${\gamma }_{ot})$ and destination-year (${\delta }_{dt})$ fixed effects account for time-varying multilateral resistance terms, while pair fixed effects (${\zeta }_{od})$ control for unobserved time-invariant phenomena and potential concerns with respect to endogeneity bias in the parameter estimate of the FTA variable (see Baier & Bergstrand, 2007).

In the second step, baseline trade costs for a country-pair-year and the related trade cost elasticities for our key trade agreement variables is calculated assuming a constant elasticity of substitution among varieties with $\sigma =7$,

2 $$\begin{aligned} (\hat{t}_{od}^{BSL} )^{{\left( {1 - \sigma } \right)}} & = {\mathbf{exp}}\left[ {\hat{\beta }_{1} lnDST_{od} + \hat{\beta }_{2} CTG_{od} + \hat{\beta }_{3} INT_{od} + \hat{\beta }_{4} PSA_{odt} + \hat{\beta }_{5} FTA_{odt} } \right. \\ & \quad \left. { + \hat{\beta }_{6} EIA_{odt} + \hat{\beta }_{7} CU_{odt} } \right] \\ \end{aligned}$$

The third step is to implement the change in trade policy for a given counterfactual scenario. Effectively, this involves a “switching on or off” of the binary PSA, FTA, EIA and/or CU variables for the relevant country-pairs under consideration. Details on the various scenarios are introduced below.

In step four, the counterfactual trade costs stemming from each counterfactual scenario in step three are estimated as

3 $$\begin{aligned} (\hat{t}_{od}^{CFL} )^{{\left( {1 - \sigma } \right)}} & = {\mathbf{exp}}\left[ {\hat{\beta }_{1} lnDST_{od}^{CFL} + \hat{\beta }_{2} CTG_{od}^{CFL} + \hat{\beta }_{3} INT_{od}^{CFL} + \hat{\beta }_{4} PSA_{od}^{CFL} + \hat{\beta }_{5} FTA_{od}^{CFL} } \right. \\ & \quad \left. { + \hat{\beta }_{6} EIA_{od}^{CFL} + \hat{\beta }_{7} CU_{od}^{CFL} } \right] \\ \end{aligned}$$

As a fifth and final step, the partial, conditional and general (full endowment) equilibrium results are calculated, as explained in detail in Yotov et al. (2016). The partial equilibrium trade volumes are a result of changes in counterfactual trade costs, while the general equilibrium results also incorporate subsequent changes in multilateral resistance terms, output and expenditure stemming from the counterfactual trade costs. Note that the model explicitly accounts for both intranational and international trade flows, such that all counterfactual scenarios induce a change in trade policy and therefore trade costs on international relative to intranational trade.

We calculate *the general equilibrium trade effects* of *five scenarios presented below.* Table 4 in the Appendix presents a detailed overview of these scenarios and a comparison of the baseline and counterfactual FTA, EIA and CU variables for the relevant country-pairs.

(I) *Brexit with cooperation agreement* Here, the baseline situation reflects the pre-Brexit situation where FTA, EIA and CU are all 1 for the country-pairs in which the United Kingdom is importer (exporter) and other EU members are exporters (importers). The counterfactual scenario is one in which the UK no longer is an EU member: the EIA and CU variables are switched from 1 to 0 for all UK-EU country pairs. Given that Brexit implies that the UK also leaves all trade agreements with non-EU countries, the trade agreement variables are switched from 1 to 0 for all UK-EU FTA partner pairs (also see below). However, the FTA variable remains 1 for all UK-EU country-pairs, reflecting the presence of the EU-UK Trade and Cooperation Agreement.^6^
(II) *Current FTAs between the United Kingdom and non-EU countries* Before Brexit, all the United Kingdom’s trade agreements with non-EU countries were arranged through agreements that applied at an EU level. This means that in a post-Brexit situation, the UK would not have a single trade deal with any country in the world other than its membership of the World Trade Organization. Signing trade deals with non-EU countries has therefore been of particular importance to the UK government to ensure a certain degree of continuity of trade relations with foreign partners. At the time of writing, the UK has reproduced several of the trade deals that formerly applied at an EU level with various countries around the world; these agreements have either already been fully ratified or are being applied provisionally.^7^ We will analyze the impact of Brexit (see above) in conjunction with the new major FTAs that the UK has successfully implemented with the economies in our dataset: Canada, Japan, Korea, Mexico, Norway, Switzerland, and Turkey. Hence, the FTA variable switches from 0 to 1 for all UK-FTA partner country-pairs.^8^
(III) *An Anglo-American FTA* Proponents of Brexit have long argued that a priority of a regained independent British trade policy would be to sign a trade deal with the United States, reflecting their most special relationship. We therefore estimate the potential trade impact of a post-Brexit Anglo-American trade deal on the UK, US and the UK's current non-EU FTA partners, while noting that the prospects of such an agreement materializing in the near future are not favorable given the July 1, 2021 expiration of the Trade Promotion Authority which the Biden administration has not yet sought to renew.^9^
(IV) *Global Britain* In this scenario, we consider a situation in which the UK has implemented FTAs with all countries, arguably reflecting trade (policy) independence from the EU in its ultimate form. In a post-Brexit world, all countries trading with the United Kingdom do so under an FTA. Nevertheless, the EIA and CU variables are again 0 for all UK-EU country-pairs, reflecting the UK’s withdrawal from the EU.
(V) *A Kingdom Divided* While the British government has vigorously pursued a ‘Global Britain’ strategy, domestic concerns arise about the unity of the United Kingdom. In our fifth scenario, we therefore ask how a decision by one of the devolved nations—Northern Ireland, Scotland, or Wales—to leave the United Kingdom and sign an FTA with the European Union might impact trade in the United Kingdom and beyond. Alternatively, the devolved nations might go even a step further and fully rejoin the EU. We examine the cases of a Scottish (Scoxit), Northern-Irish (Nirexit) and Welsh exit (Welxit) from the United Kingdom in turn. In these respective situations, the FTA, EIA and CU variables for all inter-regional UK pairs are switched from 1 to 0 to reflect secession.^10^ We then analyze the consequences of independence followed by an FTA between the newly independent nation and the UK, or an FTA with the EU. In these cases, the FTA variables for the relevant devolved nation as importer (exporter) and FTA partners as exporters (importers) are set to 1. Finally, rejoining the EU as full-fledged member is implemented by switching the FTA, EIA and CU variables from 0 to 1 for all country-pairs involving the relevant devolved nation as importer (exporter) and EU members as exporters (importer).

## Data

Our main datasets are the United States International Trade Commission (USITC)’s Gravity Portal, with bilateral (aggregated) trade data from the ITPD-E dataset (Borchert et al., 2021) and relevant time-invariant country-pair information such as bilateral distance (*DST*) and contiguity (*CTG*), and time-varying FTA membership coming from the DGD (Gurevich & Herman, 2018). The time-invariant *INT* variable is 1 for all international flows and 0 for intranational flows. Following the recommendation in Yotov et al. (2016), we use 4-year intervals (2002, 2006, 2010 and 2014) to estimate step 1–2 as discussed in the previous section, and 2014 as the most recently available year in the dataset for step 3–5. Conveniently, this selection of years avoids the 2008–09 Great Trade Collapse. We focus on 43 countries together accounting for about 90% of world trade; this helps ensure convergence of the Poisson pseudo-maximum likelihood (PPML) estimator and facilitates mapping to regional input–output data, explained below. A full list of countries is provided in Table 2. Descriptive statistics are presented in Appendix Table 5.

**Table 2:** General equilibrium effects on trade for Scenario I–IV (% change)

	(1)	(2)	(3)	(4)
	Scenario I	Scenario II	Scenario III	Scenario IV
	Brexit	Non-EU FTAs	Non-EU FTAS & US FTA	Global Britain
*Panel A: UK & EU27*				
GBR	− 40.18	− 36.41	− 31.84	− 26.52
AUT	− 0.80	− 0.81	− 0.81	− 0.83
BEL	− 2.46	− 2.47	− 2.48	− 2.52
BGR	− 1.18	− 1.20	− 1.20	− 1.25
CYP	− 5.33	− 5.36	− 5.38	− 5.49
CZE	− 1.09	− 1.10	− 1.10	− 1.13
DEU	− 2.11	− 2.12	− 2.13	− 2.17
DNK	− 2.51	− 2.53	− 2.54	− 2.59
ESP	− 3.11	− 3.13	− 3.14	− 3.19
EST	− 0.99	− 1.00	− 1.00	− 1.03
FIN	− 1.52	− 1.53	− 1.54	− 1.59
FRA	− 2.77	− 2.79	− 2.80	− 2.85
GRC	− 2.86	− 2.88	− 2.89	− 2.95
HRV	− 0.86	− 0.86	− 0.87	− 0.91
HUN	− 1.05	− 1.05	− 1.06	− 1.08
IRL	− 4.70	− 4.73	− 4.75	− 4.81
ITA	− 1.97	− 1.98	− 1.99	− 2.03
LTU	− 1.44	− 1.45	− 1.45	− 1.50
LUX	− 2.38	− 2.40	− 2.41	− 2.44
LVA	− 2.19	− 2.21	− 2.21	− 2.26
MLT	− 4.56	− 4.60	− 4.61	− 4.69
NLD	− 2.83	− 2.85	− 2.86	− 2.91
POL	− 1.62	− 1.64	− 1.64	− 1.68
PRT	− 2.70	− 2.72	− 2.73	− 2.77
ROU	− 1.38	− 1.40	− 1.40	− 1.44
SVK	− 0.81	− 0.82	− 0.82	− 0.85
SVN	− 0.80	− 0.80	− 0.81	0.20
SWE	− 2.43	− 2.45	− 2.46	− 2.53
*Panel B: Current non-EU FTA partners*				
CAN	0.23	0.63	0.61	0.59
CHE	− 0.29	0.47	0.46	0.41
JPN	0.29	0.87	0.86	0.79
KOR	− 0.41	− 0.25	− 0.25	0.14
MEX	− 0.12	− 0.05	− 0.06	0.13
NOR	− 4.11	− 2.78	− 2.80	0.58
TUR	− 0.83	0.11	0.10	1.51
*Panel C: Prospective non-EU FTA partners in dataset*				
AUS	0.33	0.32	0.31	0.94
BRA	0.29	0.28	0.28	0.72
CHN	0.20	0.20	0.20	0.47
IDN	0.17	0.17	0.17	0.34
IND	0.40	0.37	0.36	1.19
RUS	0.28	0.27	0.26	0.69
TWN	0.17	0.17	0.17	0.35
USA	0.49	0.46	1.49	1.42

In order to conduct our analyses for England, Northern Ireland, Scotland and Wales (scenario V, described above), one would ideally use high-quality interregional, bilateral trade data. While these data are—surprisingly—not available at the required level of detail for the United Kingdom (Figus et al., 2023), regional input–output tables can be used to estimate interregional trade for the UK (Greig et al., 2020) or European Union (Thissen et al., 2018). We obtain measures of final consumption expenditure by household, non-profit organizations and governments at the NUTS2 regional level for the full sample of 43 countries and aggregate these back up to the national level for all nations except the United Kingdom, for which we aggregate up to England, Northern Ireland, Scotland and Wales.^11^ While these input–output based trade measures are not entirely consistent with more advanced value-added trade metrics that have been widely adopted to study global supply-chain trade at a *national* level [e.g. Costinot and Rodriguez-Claire (2014), Koopman et al. (2014)], we propose that our measures can at least serve as a proxy for regional trade data when transaction-based official trade records are not available.

We expand the country-level gravity dataset to include England, Northern Ireland, Scotland and Wales (henceforth referred to as the “UK regions”) as follows. First, all observations involving the United Kingdom as importer, exporter, or both importer and exporter simultaneously, are linked to one of the four UK regions. Second, we adjust the national-level trade flows by weighting these with relevant regional weights derived from the input–output metrics described above. A UK region’s exports to non-UK countries are calculated as the level of UK exports to the importer, weighted by the importer’s final demand for imports from that UK region as a share of the importer’s final demand for imports from the UK overall. Similarly, a country’s exports to a UK region are calculated as the country’s exports to the UK, weighted by the UK region’s share of final demand for UK imports from the exporter. For inter- and intraregional trade flows, we weight the UK intranational trade flow by the importing UK region’s final demand from the exporting UK region over total UK final demand for imports from all UK regions. Third, the distance matrix is accordingly adjusted by updating the UK regions’ geographic coordinates in terms of latitude and longitude and recalculating the great-circle distances for all importer-exporter pairs (Picard, 2010).^12^ Fourth, the contiguity variable is updated to reflect that the UK regions do not share a common border with any other region or country, with the exception of the borders between Northern Ireland and Ireland, England and Scotland, and England and Wales, respectively. Finally, the FTAs of the UK are assumed to uniformly apply to England and the three devolved nations in the baseline situation.

## Results

### Brexit, Alternative FTAs and International Trade

We first consider how Brexit will affect international trade in general equilibrium.^13^ In line with earlier results (e.g., Brakman et al., 2018) and consistent with the broader literature (see Dhingra et al., 2017), Table 2, panel A, column 1 shows that the UK’s withdrawal from the EU brings about a significant decline in international trade for the UK (around − 40%), as well as substantial decreases in trade across the EU members where Ireland, Cyprus and Malta face the strongest decline of around 5%. These results are generally in line with our prior expectations; interestingly, Brakman et al. (2018) find that when supply-chain trade is taken into account, Ireland, Belgium, The Netherlands, Germany and France will be most severely affected by Brexit. The difference in these results stems from the fact that the present paper relies on gross exports as a measure of international trade, rather than trade in value-added.

Once the UK has withdrawn from the EU, Table 2, column 2 shows how the signing of new FTAs by the UK government will affect international trade for the UK and its former EU members. For the latter set of countries, UK FTAs with third countries will have very limited trade diverting effects; Brexit induces the most significant decline in EU trade. For the UK, additional FTAs in the wake of Brexit still reduces overall trade by around 36%, which only softens the blow of Brexit on the UK by about 4 percentage points. In addition, establishing an FTA with the United States would be of significant economic importance to the UK, further limiting the Brexit-induced trade loss to about 31% as per Table 2, column 3. Yet, “the world is not enough” for the United Kingdom to fully undo the trade loss inflicted by its withdrawal from the EU: Table 2, column 4 shows that even if the UK were to succeed in implementing trade deals with all countries in our dataset except EU members, the decline in trade still amount to an impressive 26%. Together, these findings reflect the paradoxical outcome that the United Kingdom relies entirely on the members of the European Union on implementing a trade deal that can offset the self-inflicted economic wounds of Brexit (also see Brakman et al., 2018).

How will non-EU countries be affected by Brexit and the introduction of new FTAs with the UK? Table 2, panel B and C show the general equilibrium changes in trade for all (potential) non-EU FTA partners in our dataset. Four results stand out. First, Brexit itself induces relatively small changes in trade with non-EU countries, the largest negative shock being experienced by Norway. Second, we confirm positive (yet marginal) effects of the current non-EU FTAs that the UK has signed with Canada, Japan, Switzerland and Turkey, while gains from the deals with Korea and Mexico are not in line with our expectations. Third, our results suggest that an Anglo-American FTA would indeed be beneficial from a trade perspective, not only for the United Kingdom as already discussed above, but also for the United States with an increase in trade of about 1%. Finally, while a Global Britain strategy would not help the United Kingdom to entirely compensate for the loss in Brexit-induced trade, potential UK partners which could stand to gain from an FTA with the UK include Australia and India (1% each). The economic gains from an FTA with China and other countries are limited.

### Brexit, a Break-Up of the UK and International Trade

We now turn to the question of how Scottish, Northern-Irish or Welsh secession from the United Kingdom and their other possible trade policy options will affect these nations’ trade. Table 3 presents our general equilibrium trade effects of Scenario V for the nations of the United Kingdom.

**Table 3:** General equilibrium effects on trade for Scenario V (% change)

(1)	(2)	(3)	(4)	(5)	(6)	(7)	(8)
	Secession & no EU membership				$\leftarrow$ Brexit $\to$	Secession &EU membership	
*Panel A: NIREXIT*							
England	− 24.5	− 25.6	− 25.0	− 26.0	− 24.8	− 26.6	− 27.6
Northern Ireland	− 14.9	− 13.3	− 11.5	− 10.1	− 9.2	− 0.9	0.0
Scotland	− 15.0	− 15.7	− 15.3	− 16.0	− 15.2	− 16.3	− 16.9
Wales	− 12.4	− 12.4	− 12.4	− 12.4	− 12.4	− 12.5	− 12.5
*Panel B: SCOXIT*							
England	− 24.8	− 26.1	− 25.2	− 26.6	− 24.8	− 27.0	− 28.3
Northern Ireland	− 9.2	− 9.5	− 9.3	− 9.7	− 9.2	− 9.7	− 10.1
Scotland	− 25.7	− 22.5	− 20.1	− 17.0	− 15.2	− 2.3	0.0
Wales	− 12.4	− 12.4	− 12.4	− 12.4	− 12.4	− 12.5	− 12.5
*Panel C: WELXIT*							
England	− 24.5	− 25.9	− 25.0	− 26.4	− 24.8	− 27.2	− 28.4
Northern Ireland	− 9.2	− 9.2	− 9.2	− 9.2	− 9.2	− 9.3	− 9.3
Scotland	− 15.1	− 15.2	− 15.2	− 15.2	− 15.2	− 15.3	− 15.3
Wales	− 20.3	− 17.5	− 15.6	− 13.1	− 12.4	− 1.6	0.0
UK-EU FTA	Yes	Yes	Yes	Yes	Yes	Yes	Yes
Secession	Yes	Yes	Yes	Yes	No	Yes	Yes
FTA with UK	Yes	No	Yes	No	No	Yes	No
FTA with EU	No	No	Yes	Yes	No	Yes	Yes
EU member	No	No	No	No	No	Yes	Yes

Table 3 presents the general equilibrium effects of Brexit, a devolved nation’s secession and the prospects of its potential FTA’s with either the rest of the UK, a renewed FTA with the EU, or renewed membership of the EU. Panel A shows the results induced by a Northern-Irish secession, Panel B presents the consequences of Scottish independence, and Panel C considers the case of Welxit.

In all panels, the scenarios are ranked from left to right, in order of magnitude for the relevant devolved nation that seeks independence. In all scenarios, we assume that the UK as a whole, or at least those countries that remain in the UK, maintain an FTA with the European Union.^14^ We consider column (6) as the ‘baseline’ situation reflecting the general equilibrium effects of Brexit in the UK. Our estimates indicate that England suffers a 25 percent decrease in its trade due to Brexit; trade performance decreases for Northern Ireland, Scotland and Wales by 9, 15 and 12 percent, respectively.

The next step is to evaluate how any of these devolved nations’ independence from the UK impacts their trade. Columns (2)–(5) show that independence will bring about an even larger decrease in trade. For example, the combined effect of Brexit and Scoxit would reduce Scotland’s trade by about 23% in general equilibrium. From the vantage point of the current Brexit situation, the prospects of secession are also unfavorable for Wales (decrease of about 18 percent) and Northern Ireland (decrease of about 13 percent).

Our results suggest that independence in itself is not an appealing business from a trade perspective, as disrupting a devolved nation’s FTA with the remainder of its former UK counterparts puts it in further isolation in terms of trade policy. Qualitatively our results for Scotland are consistent with those of Huang et al. (2021) who also find that Scotland stands out in trade terms.

While our results do not suggest promising trade outcomes from independence itself, the prospects change once we evaluate independence conditional on regained economic integration with the European Union through EU membership in columns (7) and (8). In all cases, we find that independence together with rejoining the EU as full-fledged member would undo the trade damage imposed by Brexit, but the potential to generate additional trade creation beyond the “break-even point” is very limited. Our results also suggest that in these cases, England would in suffer an additional loss in trade of about 2–4 percentage points relative to the current Brexit context.

The necessary caution is advised when interpreting our results. Even if a devolved nation succeeds in gaining independence and signing a trade deal with the EU—the geopolitical feasibility of such an outcome is not set in stone—the extent to which an FTA would result in gains from trade depends on the depth of the agreement and the extent to which acceding nations seek to re-integrate their markets for goods, services, labor and capital with the European Single Market. Yet, overall, our results suggest that even full-fledged EU membership will only be sufficient to undo the economic damage of Brexit and secession from the UK.

### Mapping the Distance Equivalents of the Trade Impact: the UK Becomes Greenland

To visualize the importance of trade barriers and the role these barriers play in changing the global trade landscape, we now consider a complementary approach to illustrating the impact of Brexit and independence of the devolved nations. In the following simulations, we consider how counterfactual changes in *bilateral distance* impacts trade, rather than changes in FTAs induced by various EXIT scenarios. This done by taking Eq. (1) and replacing the time-varying multilateral resistance terms with an origin-specific and destination-specific fixed effect; the dyadic fixed effect is dropped. These modifications are motivated by the fact that we are now no longer interested in changes in the time-variant independent trade policy variables of interest, but only in a counterfactual change in the time-invariant distance variable. The purpose of this didactic exercise is to simply provide an intuitive, complementary illustration of the impact of Brexit on trade through changes in trade costs if these were induced by changes in geography, rather than trade policy.

**Fig. 3:**
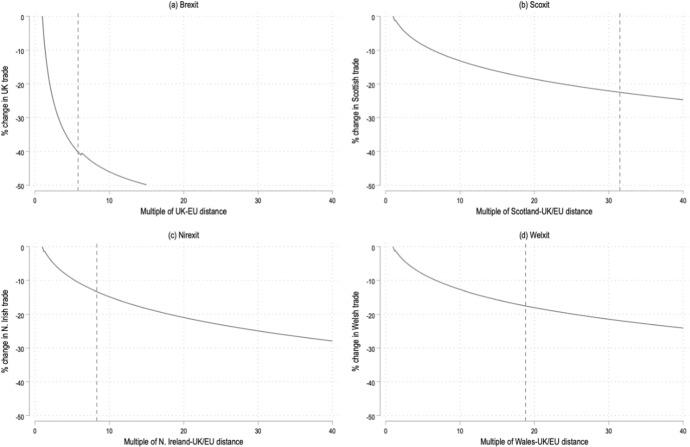
General equilibrium trade effect equivalents in terms of geographic distance. *Notes:* In panel (**a**), the dashed line indicates by how much geographic distance between the UK and EU would have to increase (without Brexit) to reach a similar decline in trade resulting from Brexit (holding geographic distance constant); in panel (**b**), the dashed line shows that the distance between Scotland and the UK/EU would have to increase by close to a factor 31.5 (without a Scoxit) to approximate a Scoxit-induced decrease in trade (holding geographic distance unchanged), etc.

In the scenarios that follow, we change the bilateral distance by a multiple of 1 or more and calculate the associated change in that country or region’s trade. To be precise, in the case of Brexit, only the distance between the UK and its EU counterparts is changed; all other distances remain fixed. It is as if one would geographically move the UK farther away from the EU for all UK-EU trade, but not for all other trading relationships. Figure 3, panel (a) shows how the general-equilibrium trade effects of Brexit (− 40.2 percent, see Table 2) are approximated by a 5.8-fold increase of the geographic (great-circle) distance between the UK and EU; the equivalence of shifting, for example, the UK all the way to Greenland.

Next, we calculate by how much geographic distance between Scotland and the remainder of the UK and EU member states would have to increase to approximate our trade effects of Scottish secession (− 22.5 percent, see Table 3). Figure 3, panel (b) shows that this would require increasing the relative distance by a magnitude of 31.5, moving Scotland all the way to Australia relative to the UK and EU. Panel (c) shows that a Nirexit would require shifting Northern Ireland to Russia to approximate the 13.3 percent loss in trade, reflecting an increase in the relative distance by a magnitude of 8.3. Finally, the trade effects of a Welxit (− 17.5 per cent per Table 3) could also be obtained by shifting Wales to Syria, indicative of the required increase in distance by a factor 18.8.

## Summary and Conclusion

We estimated the trade effects of Brexit and various trade arrangements for not only the UK, but also for its main trading partners in- and outside the EU. In addition, we used a gravity model approach to estimate the trade impact of a break-up of the UK where we allow for the possibility that the newly ‘independent’ nations that exit the UK sign up to an FTA with the EU, or to even fully rejoin the EU. To visualize our results, we also presented the (bilateral) distance equivalent effects of the changes in the trade scenarios that were considered. This analysis takes place against the background that Brexit has actually materialized and that at least for now international trade for the UK seems markedly lower than it would have been without Brexit. Our findings confirm the main conclusions from related studies to the effect that the UK cannot make up for the trade and hence welfare loss caused by Brexit by signing alternative FTAs. While Brexit and devolved nations’ secession would raise trade costs and thus induce more domestic consumption, the blowback despite additional FTAs on UK trade is still very substantial [see, e.g., Oberhofer and Pfaffmayr (2021)]. Also, grand schemes like ‘Global Britain’ will not succeed in doing so. In addition, independence from the UK by Scotland, Northern-Ireland or Wales would further increase economic damage for these devolved nations, but a renewed FTA with the EU by the newly ‘independent nations’ could marginally compensate for their break-up with the UK. Yet, even full-fledged EU membership would only marginally undo the economic damage of Brexit and secession as far as trade is concerned. To put our findings in (visual) perspective we derived the distance equivalent effects of both Brexit and the break-up of the UK: in distance terms our gravity estimations imply for instance (or most notably) that the UK would become Greenland so as to get perspective on the size of the negative impact of Brexit in trade terms.

There are a number of ways as to how our analysis could be extended. First of all, one could use more refined trade data. We stick to gross trade data in the present paper so as to be able to perform the intra-UK analysis. The use of for instance value-added trade data, like in Brakman et al. (2018), would allow research to be more specific on the trade impact and to take the relevance of value chains into account. Secondly, a more in-depth analysis of trade barriers would be useful. Certainly, in the case of Brexit, the real trade impact for firms and hence customers comes from non-tariff, regulatory trade barriers (see, e.g., Figus et al., 2023). Finally, a sectoral breakdown of the analysis would enable more precise predictions where the impact of Brexit or alternative FTAs will be felt most in a positive or negative way.^15^ Despite these avenues for future research and consequently limitations of the current study, and also taking into account that Brexit has come into effect since early 2020, it remains a fair conclusion to state that Brexit did and will not make much economic sense for the UK from the perspective of international trade. On the contrary, alternative FTAs clearly cannot make up for the trade and welfare losses for the UK as a whole caused by Brexit. A ‘devolved nation’ scenario might ultimately enable parts of the UK to undo the negative Brexit impact by regaining membership of EU, but only at the cost of leaving the UK. More generally, and in terms of possible political economic geography implications, our analysis shows how *dis*integration at the national level could actually trigger or strengthen disintegration processes at the within-country regional level if regions differ in their preferences for international integration and national unity. Regions put relatively more weight on the former than on the latter may actually feel bolstered to go their own way. In Brexit parlor, ‘Global Britain’ cannot offset the cost of the EU-UK divorce but it may pave the way to a break-up of the United Kingdom.

## Appendix

Tables 4, 5 and 6.

**Table 4:** Implementation of counterfactual scenarios

Scenario	Variables						Pairs affected
	FTA		EIA		CU		
	Baseline	Counterfactual	Baseline	Counterfactual	Baseline	Counterfactual	
I	1	1	1	0	1	0	All UK-EU pairs
	1	0	0	0	0	0	All UK-EU FTA partner pairs
II	1	1	1	0	1	0	All UK-EU pairs
	1	1	0	0	0	0	All UK-FTA partner pairs
III	1	1	1	0	1	0	All UK-EU pairs
	1	1	0	0	0	0	All UK-FTA partner pairs
	0	1	0	0	0	0	All UK-USA pairs
IV	1	1	1	0	1	0	All UK-EU pairs
	0/1	1	0	0	0	0	All UK-non EU pairs
V	1	0	1	0	1	0	All EXIT-Rest of UK pairs (Secession)^1^
	1	1	1	0	1	0	All EXIT-Rest of UK pairs (Secession + UK-FTA)
	1	1	1	0	1	0	All EXIT-EU pairs (Secession + EU-FTA)
	1	1	1	1	1	1	All EXIT-EU pairs (Secession + EU membership)

Table displays changes in FTA, EIA and/or CU variables from baseline to counterfactual scenarios as described in the main text. For Scenario V, “EXIT” represents Scotland, Northern Ireland, or Wales for the respective cases of Scoxit, Nirexit and Welxit

^1^For the sake of simplicity, we assume that the seceding nation will be able to maintain its set of FTAs that do not involve the EU or the rest of the UK through transitional arrangements

**Table 5:** Descriptive statistics

Variable	Obs	Mean	Std. dev	Min	Max
*Country-level data for Scenarios I–IV:*					
year	7,396	2008	4.472	2002	2014
ln_DIST	7,396	7.999	1.146	0	9.825
CNTG	7,396	0.058	0.235	0	1
INT	7,396	0.023	0.151	0	1
PSA	7,396	0.021	0.142	0	1
FTA	7,396	0.498	0.500	0	1
EIA	7,396	0.447	0.497	0	1
CU	7,396	0.347	0.476	0	1
*Country-level and UK regional data for Scenario V:*					
year	8,464	2008	4.472	2002	2014
ln_DIST	8,464	7.963	1.141	0	9.825
CNTG	8,464	0.053	0.224	0	1
INT	8,464	0.022	0.146	0	1
PSA	8,464	0.018	0.133	0	1
FTA	8,464	0.523	0.499	0	1
EIA	8,464	0.471	0.499	0	1
CU	8,464	0.373	0.484	0	1

**Table 6:** Baseline gravity estimates

	(1)	(2)	(3)	(4)
	Only countries (for Scenarios I–IV)		Also incl. UK regions (for Scenario V)	
log(Distance)	− 0.533***	–	− 0.199**	–
	(0.0981)		(0.0752)	
Contiguity	0.601***	–	0.685***	–
	(0.124)		(0.122)	
International	3.600***	–	4.257***	–
	(0.267)		(0.224)	
Partial Scope Agreement	0.0937	0.223*	0.0956	0.232*
	(0.340)	(0.111)	(0.373)	(0.104)
Free Trade Agreement	− 0.0972	0.150*	0.170	0.158*
	(0.126)	(0.098)	(0.137)	(0.094)
Customs Union	− 0.140	0.191***	0.296*	0.213***
	(0.105)	(0.034)	(0.141)	(0.031)
Economic Integration Agreement	0.147	0.310***	0.116	0.307***
	(0.118)	(0.096)	(0.162)	(0.092)
Constant	7.964***	7.384***	5.007***	7.105***
	(0.892)	(0.007)	(0.694)	(0.009)
N	7,396	7,396	8,464	8,464
Importer-Year FE	Yes	Yes	Yes	Yes
Exporter-Year FE	Yes	Yes	Yes	Yes
Importer-Exporter FE	No	Yes	No	Yes

Baseline gravity estimates for Eq. (1). Standard errors (clustered by country-pair) in parentheses. **p* < 0.05, ***p* < 0.01, ****p* < 0.001

## Footnotes

1 For detailed data see: https://www.ons.gov.uk/businessindustryandtrade/internationaltrade/articles/theimpactsofeuexitandthecoronavirusonuktradeingoods/2021-05-25#main-points.

2 The interested reader can find summaries of the most relevant papers on the impact of Brexit on trade and income on the CEPR related VOXeu website: https://voxeu.org/search/node/Brexit. See also Dhingra and Sampson (2022) for a recent survey.

3 Huang et al. (2021) provide detailed trade estimates for Scottish independence. Besides Scotland we also study independence of Wales and Northern-Ireland.

4 While Brexit naturally affects EU27 members – as our ex-ante trade effects for Brexit in Sect. 5 also show—the Withdrawal Agreement contains a transition period for February 1 to December 31, 2020, ensuring continued frictionless trade between the UK and EU while the EU-UK Trade and Cooperation Agreement were being negotiated (https://ec.europa.eu/info/relations-united-kingdom/eu-uk-withdrawal-agreement_en). As such, we propose that it is reasonable to assume that the observed declines in EU27 trade in 2020 are largely, if not entirely, related to the pandemic, rather than the UK’s withdrawal from the EU.

5 Trade agreements vary in their design and economic outcomes (Baier et al., 2019; Kohl, 2014; Kohl et al., 2016). For an analysis of heterogeneous trade agreements in the context of Brexit, see, e.g., Felbermayr et al. (2022).

6 In unreported robustness checks, we also calculate the general equilibrium trade effects in the event of a “Hard Brexit” where the FTA variable also switches to 0 for all scenarios under consideration. While these effects are plausibly larger in order of magnitude, our main conclusions are qualitatively similar. These results of scenarios without an EU-UK FTA are available from the authors upon request. Also note that the UK and EU do not participate in PSAs for the years under consideration, which is why the PSA variable remains unchanged in the counterfactual scenarios.

7 For an up-to-date list of the UK’s non-EU trade deals, see https://www.gov.uk/guidance/uk-trade-agreements-with-non-eu-countries.

8 Note that in scenario I, we already assume that the UK maintains an FTA with the EU. We continue this assumption throughout scenario I–V.

9 See, for example, https://www.politico.com/newsletters/weekly-trade/2021/06/28/say-goodbye-to-trade-promotion-authority-796173.

10 Note that intraregional and intranational trade, i.e., “trade with self”, is never subject to a trade agreement so that the PSA, FTA, EIA and CU variables are always zero in cases when the importer is identical to the exporter.

11 This so-called PBL EUREGIO database (2000–2010) is available at https://data.overheid.nl/dataset/pbl-euregio-database-2000-2010. Values for 2010 are assumed to be constant for 2014.

12 Latitudes and longitudes were obtained from OpenStreeMap.org. Intraregional distances remain unaffected and consistent with those provided by Gurevich and Herman (2018); for the four UK regions, the new intraregional distance value is the UK intraregional distance weighted by the devolved nation’s share of the UK’s geographic area (Office for National Statistics 2016).

13 For baseline gravity estimates for Eq. (1), see Appendix Table 6.

14 Our unreported results without an FTA between the UK and EU are quantitatively and qualitatively very similar to our reported findings, and are available from the corresponding author upon request.

15 Given the absence of official trade data for the devolved nations with the UK and the rest of the world, it is beyond the scope of this paper to construct international bilateral trade measures at the NUTS2 regional-sector level for the countries in our sample. A complementary approach is provided by Huang et al. (2021), who use the World Input–Output Database to construct their trade measures at a country-sector level. The authors use Scottish input–output matrices to decompose UK sectoral trade into a Scottish share and a ‘rest of the UK’ share. Sectoral trade is then calculated for Scotland, the rest of the UK, the EU27, and a rest-of-the-world aggregate. They found that Scotland's 2014 exports were predominantly in mining and quarrying (26%), other services (22%), food products, beverages and tobacco (12%) and financial and insurance services (9%).
